# Wide-Oblique-Incident-Angle Stable Polarization-Insensitive Ultra-Wideband Metamaterial Perfect Absorber for Visible Optical Wavelength Applications

**DOI:** 10.3390/ma15062201

**Published:** 2022-03-16

**Authors:** Mohammad Lutful Hakim, Touhidul Alam, Md. Shabiul Islam, M. Salaheldeen M., Sami H. A. Almalki, Mohd Hafiz Baharuddin, Haitham Alsaif, Mohammad Tariqul Islam

**Affiliations:** 1 Pusat Sains Ankasa (ANGKASA), Institut Perubahan Iklim, Universiti Kebangsaan Malaysia, Bangi 43600, Selangor, Malaysia; p108762@siswa.ukm.edu.my; 2 Department of CSE, International Islamic University Chittagong (IIUC), Kumira, Chattogram 4318, Bangladesh; 3 Faculty of Engineering (FOE), Multimedia University, Persiaran Multimedia, Cyberjaya 63100, Selangor, Malaysia; shabiul.islam@mmu.edu.my; 4 Department of Electrical Engineering, Faculty of Energy Engineering, Aswan University, Aswan 81528, Egypt; mohamedsalah40@hotmail.com; 5 Department of Electrical Engineering, College of Engineering, Taif University, P.O. Box 11099, Taif 21944, Saudi Arabia; s.h.almalki@tu.edu.sa; 6 Department of Electrical, Electronic and Systems Engineering, Faculty of Engineering and Built Environment, Universiti Kebangsaan Malaysia, Bangi 43600, Selangor, Malaysia; hafizb@ukm.edu.my; 7 Electrical Engineering Department, College of Engineering, University of Ha’il, Ha’il 81481, Saudi Arabia; h.alsaif@uoh.edu.sa

**Keywords:** visible optical wavelength, metamaterial absorber, polarization-insensitive, oblique incident stability, bendable

## Abstract

Metamaterial absorbers are very attractive due to their significant absorption behavior at optical wavelengths, which can be implemented for energy harvesting, plasmonic sensors, imaging, optical modulators, photovoltaic detectors, etc. This paper presents a numerical study of an ultra-wide-band double square ring (DSR) metamaterial absorber (MMA) for the complete visible optical wavelength region, which is designed with a three-layer (tungsten-silicon dioxide-tungsten) substrate material. Due to the symmetricity, a polarization-insensitive absorption is obtained for both transverse electric (TE) and transverse magnetic (TM) modes by simulation. An absorption above 92.2% and an average absorption of 97% are achieved in the visible optical wavelength region. A peak absorption of 99.99% is achieved at 521.83 nm. A wide range of oblique incident angle stabilities is found for stable absorption properties. A similar absorption is found for different banding angles, which may occur due to external forces during the installation of the absorber. The absorption is calculated by the interference theory (IT) model, and the polarization conversion ratio (PCR) is also validated to verify the perfect MMA. The electric field and magnetic field of the structure analysis are performed to understand the absorption property of the MMA. The presented MMA may be used in various applications such as solar cells, light detection, the biomedical field, sensors, and imaging.

## 1. Introduction

Veselago first demonstrated the unusual electromagnetic properties such as negative permittivity or permeability of complex materials [1], which is known as metamaterials (MMs) [2]. The characteristics of MMs depend on its physical structure, not its chemical composition, which makes it suitable for use in various applications such as absorbers [3], filters [4], antennae [5], imaging [6], invisible clocks [7], sensors [8], polarization converters [9], and lenses [10]. Landy first introduced the metamaterial perfect absorber (MPA) [11], which has opened a new research area due to its extensive use in different applications such as energy harvesting [12], solar cells [13], photodetectors [14], and thermal emitters. [15]. Researchers are currently conducting extensive research on the MPA design for application in the infrared, THz, and microwave frequencies, and they are achieving low-loss devices through structural optimization [16,17]. In addition, solar energy harvesting using the MPA of total visible light has drawn research attention [18,19,20]. Appropriate electric and magnetic resonance engineering can achieve uniform absorption properties. Most researchers have shown different types of MPA structures with limited absorption properties such as limited frequency and low absorption level. Only a few studies have been found on the full visible optical wavelength region. The visible wavelength range is 380–750 nm, which corresponds to a frequency spectrum of 400–790 THz. Polarization-insensitive properties are essential for absorber design because of the unique absorption rate at different polarization incident angles [8,21]. Moreover, a broad level of incident angle stability is required for efficient absorption rate [22].

In [23], a Cu, Si_3_N_4_, and Si material-based four-layer optical wavelength absorber was designed for 400–700 nm, where the lowest absorption was about 80% with a peak absorption level of 97%. In [24], an absorption above 83% was found in the visible optical wavelength region from 370 to 880 nm, and the peak absorption value reached 92% by using Au and SiO_2_ materials. In [25], a small-size Ag and SiO_2_-based absorber was presented for 300–700 nm, where the absorption bandwidth and absorption level were decreased, but the peak absorption value was increased to 98%. In addition, the polarization insensitivity was not found in [23,24,25] and the oblique incident angle stability was also not presented. In [22], a three-layered (tungsten-silicon dioxide-tungsten) metamaterial optical region absorber was presented, where an absorption bandwidth above 91.24% was achieved from 389.34 to 697.19 nm with a peak absorption of 99.99%. The polarization insensitivity and 60° incident angle stability were found at absorption levels up to 70%. In [26], an Au and Si-based absorber was proposed with increased incident angle stability up to 65°. However, the absorption level decreased to 80%. A Ni and Si-based absorber was proposed for an absorption level above 90% at wavelengths from 400 to 700 nm [27]. After analyzing the previous research, it is clear that an MMA that is polarization-insensitive and has oblique incident angle stability with a high-absorption-level MMA for the entire wavelength (380–750 nm) is highly desirable for visible optical metamaterial absorber applications.

This paper presents a three-layer MMA of ultra-wide absorption bandwidth from 380 to 750 nm for visible optical wavelengths. The polarization conversion ratio (PCR) value was analyzed to verify the proposed design as an absorber. Absorption properties at different polarization angles and oblique incident angles were analyzed for both TE and TM modes, which showed unique absorption properties for both modes. The proposed MMA achieved an absorption above 92.2% at the operating wavelength with an average and peak absorption of 97.2% and 99.99%, respectively.

## 2. Absorber Design

**Figure 1:**
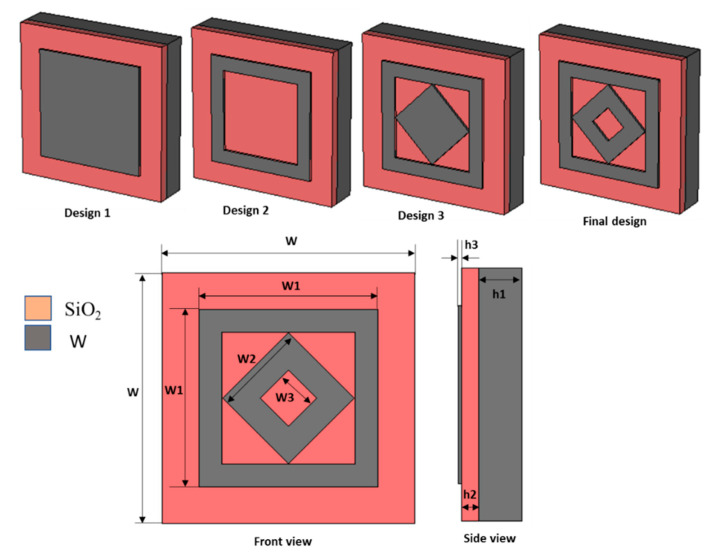
Design of proposed MMA of visible optical wavelength application.

Figure 1 shows the three-layer metamaterial absorber structure, where the middle layer uses silicon dioxide (SiO_2_) as the dielectric substrate and tungsten (W) as a patch and ground on both sides of the SiO_2_. The material properties of SiO_2_ and W were considered from [28]. The purpose of using W in the patch and ground of this design was due to the high intrinsic loss and impedance matching feature with free space in optical wavelength applications. Therefore, this leads to an increased absorption behavior by offering low reflection and transmission properties [29]. The SiO_2_ was selected as a dielectric substrate due to its lossless property at optical wavelengths and large negative part of permittivity in the whole optical region instated of the large imaginary part. These features of SiO_2_ provide a good impedance matching and lead to a high and more comprehensive absorption bandwidth [30]. The proposed DSR metamaterial absorber was designed and simulated using Frequency Integration Technique (FIT)-based Computer Simulation Technology (CST) [31]. The overall design parameters are listed in Table 1. Unit cell boundary conditions on the x- and y-axis and open add space on the z-axis were applied for the simulation. The absorption property *A*(*ω*) was calculated by using Equation (1) [8].

(1) $$A(\omega )=1-{\left|S_{11}(\omega )\right|}^{2}-{\left|S_{21}(\omega )\right|}^{2}=1-R-T$$

where *S*_11_ and *S*_21_ are the reflection coefficient and transmission coefficient, respectively. *R* and *T* are the reflectance and transmittance, respectively. Due to the thick back layer, the transmission of electromagnetic waves blocks and the transmission coefficient *S*_21_ become zero.

**Figure 2:**
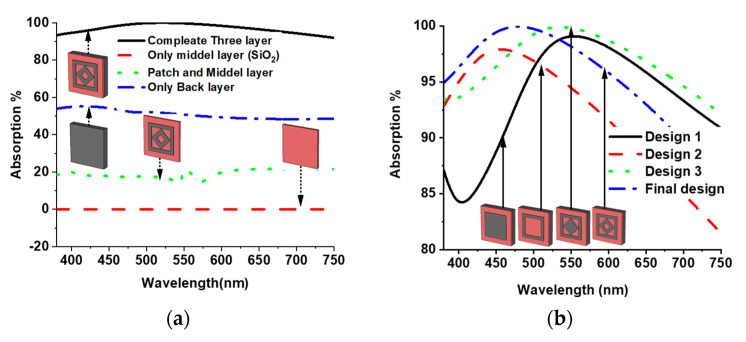
Absorption of (**a**) different layers and (**b**) design evolution.

Figure 2a presents the absorption properties for the different layers, where no absorption was observed only for the SiO_2_ dielectric layer. Due to all incident waves transmitted through the dielectric material, an absorption around 20% was realized by adding a patch on top of the dielectric, but the maximum incident wave still transmitted through the material. After that, the W base ground plane was added to prevent the transmission of the incident wave and a 97.1% average absorption level was attained in the entire visible optical wavelength region. The geometrical design of the patch also played a crucial role in achieving higher and wider absorption levels, which can be understood from the design evolution of the proposed MMA. The absorption property of the proposed MMA of various design evolutions is presented in Figure 2b. The peak absorption reached 99% at 553.63 nm, and an average absorption of 92.46% was achieved for design 1, where only a square resonator was used. In design two, a square slot was made in the patch of design 1, which increased the average absorption level to 94.46%, and the peak absorption shifted toward 495 nm. By adding another square patch inside the square ring in design 2, the average absorption increased up to 96.70% in design 3. Finally, an average absorption of 97.1% was attained by making a square slot at the center of the square plane in design 3.

**Figure 3:**
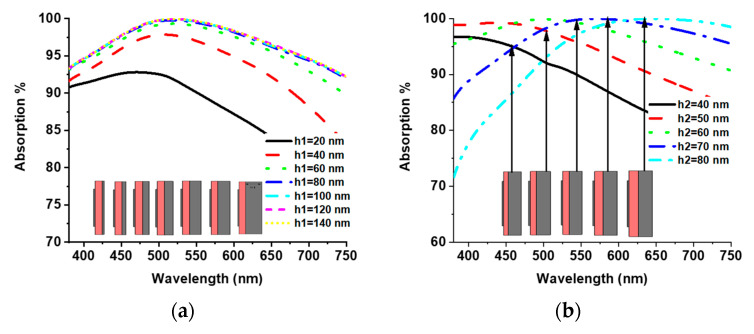

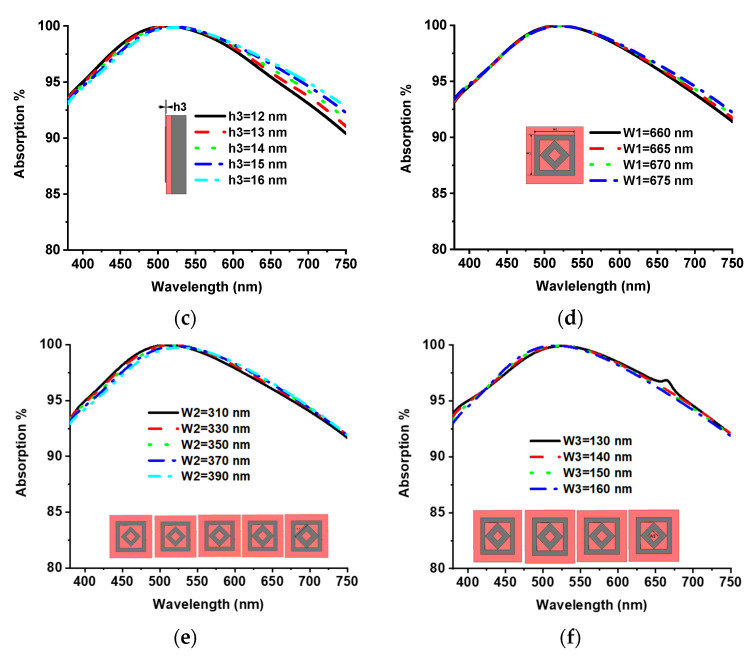
Absorption of different values: (**a**) ground plane thickness, (**b**) dielectric thickness value, (**c**) patch thickness, (**d**) outer ring length, (**e**) inner ring length, and (**f**) inner ring slot length.

The parametric analysis was also very important for understanding the absorption properties of the proposed MMA. The parameter h1 represents the thickness of W at the ground plane. The absorption property was investigated from 20 to 140 nm with a 20 nm interval for h1 and is presented in Figure 3a. The absorption level increased with h1 to 80 nm and displayed an almost unique absorption curve for higher thicknesses. The MMA design chose the h1 = 120 nm parametric value, which completely blocked the transmission and provided a higher absorption level. The dielectric thickness (SiO_2_) also significantly influenced higher absorption levels. The parameters h2 represents the dielectric thickness, and the absorption at different dielectric thicknesses is presented in Figure 3b. It is shown that the absorption value was higher in the lower optical wavelength region than the upper optical wavelength region for h2 = 40 nm and 50 nm. On the other hand, for higher thicknesses, the absorption value was higher in the upper optical wavelength region than the lower optical wavelength region. The maximum average absorption level was achieved at h2 = 60 nm. The thickness of the MMA patch was also investigated for h3. A constant absorption was found for different h3 values with slight distortion in the upper optical wavelength region. For maximum absorption level, h3 = 15 nm was settled. Figure 3d shows the impact of the outer square ring resonator from 660 to 675 nm with a 5 nm interval, where a stable absorption bandwidth was realized, and very few deformations were found in the upper wavelength region. W1 = 670 nm was chosen for the higher absorption level. The absorption at different values of the inner square ring length (W2) from 310 to 390 nm is presented in Figure 3e, which shows a slight distortion in the absorption curve. Figure 3f demonstrates the effect of inner ring slot length (W3) from 130 to 160 nm, showing a stable absorption for different values. The investigation showed that the optimized top layer parameters (h3, W1, W2, and W3) of the proposed design will provide a sound absorption level.

## 3. Results Analysis

### 3.1. Absorption Characteristics

**Figure 4:**
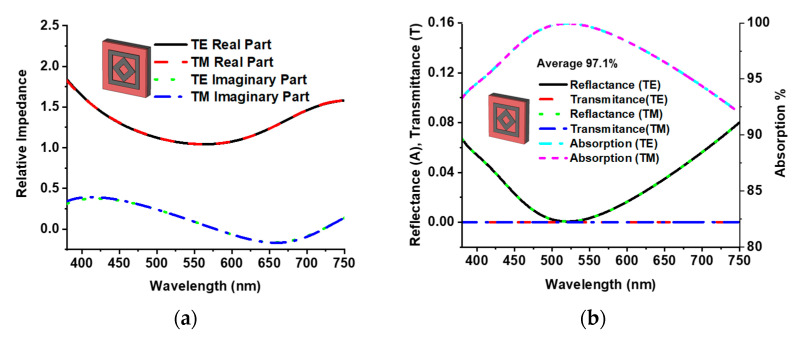
(**a**) Normalized impedance and (**b**) reflectance, transmission, and absorption plot.

The absorption characteristics depend on the impedance matching of the structure. The relative impedance (Z) of the proposed three-layer sandwich model was calculated using Equation (2) [32], and Figure 4a presents the relative impedance of both TE and TM modes. The near-unity value of the real part and near null value of the imaginary part shows that the proper effective impedance of the structure matches with the free space impedance, which offers a high absorption level.

(2) $$Z=\sqrt{{\left(1+S_{11}\right)}^{2}-S_{21}^{2}/{\left(1-S_{11}\right)}^{2}-S_{21}^{2}}=\sqrt{\mu /\varepsilon }/Z_{0}=\sqrt{\mu_{r}/\varepsilon_{r}}$$

where $\mu =\mu_{r}\mu_{0}$ and $\varepsilon =\varepsilon_{r}\varepsilon$ are the permeability and permittivity of the MMA, respectively. $\mu_{r}$ and $\varepsilon_{r}$ are the relative permeability and permittivity of the medium, respectively. $\mu_{0}$ and $\varepsilon_{0}$ are the permeability and permittivity of free space, respectively. Thus, the free space impedance is $Z_{0}=\sqrt{\mu_{0}/\varepsilon_{0}}$. The reflectance (*R*) of the TE and TM mode can be calculated by Equations (3) and (4), respectively [17]:

(3) $$R_{TE}={\left|r_{TE}\right|}^{2}={\left|\mu_{r}\cos\theta -\sqrt{n^{2}-\sin\theta }/\mu_{r}\cos\theta +\sqrt{n^{2}-\sin\theta }\right|}^{2}$$

(4) $$R_{TM}={\left|r_{TM}\right|}^{2}={\left|\varepsilon_{r}\cos\theta -\sqrt{n^{2}-\sin\theta }/\varepsilon_{r}\cos\theta +\sqrt{n^{2}-\sin\theta }\right|}^{2}$$

where *n* is the refractive index, and *θ* is the incident angle of the wave for typical incidence. Thus, Equations (3) and (4) become:

(5) $$R_{TE,TM}={\left|Z-Z_{0}/Z+Z_{0}\right|}^{2}={\left|\sqrt{\mu_{r}}-\sqrt{\varepsilon_{r}}/\sqrt{\mu_{r}}+\sqrt{\varepsilon_{r}}\right|}^{2}$$

Equation (5) represents the reflectance of the MMA, which is significantly controlled by impedance matching as well as the metamaterial. As the transmission is zero due to the back layer, absorption (*A*) can be calculated by A=1−R. The reflectance, transmittance, and absorption characteristics of TE and TM modes are illustrated in Figure 4b. The symmetrical design of the proposed MMA provides a unique absorption curve for both TE and TM modes. A peak absorption of 99.99% is realized at a wavelength of 523 nm, and an absorption above 99% appears from 474.36 to 578.75 nm. In addition, an absorption more than 92.2% is obtained for the entire visible optical wavelength region with an average absorption of 97.1%. This near-unity absorption feature of the proposed MMA can be used for solar energy harvesting at visible optical wavelengths, stealth technology, sensors, and micro-imaging technology [21].

**Figure 5:**
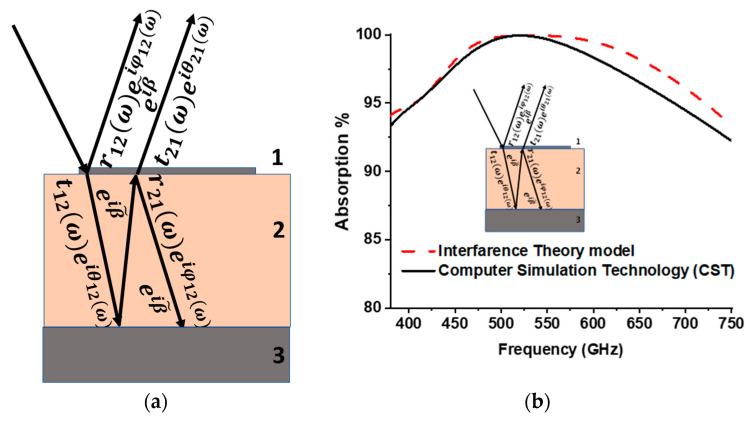
(**a**) Interference theory (IT) model, and (**b**) absorption plot of Interference Theory (IT) model and Computer Simulation Technology (CST).

Absorption characteristics can also be calculated by applying the Interference Theory (IT) model, which also explains the underlying physics of the metamaterial absorber [33,34]. The patch (layer 1) with a particular metallic pattern acts as a partial reflection surface. This layer modifies complex transmission and reflection coefficients, and the ground plane (layer 3) works as a perfect reflector. Figure 5a shows the patch between air and a dielectric substrate. The incident EM wave partially transmits and reflects the atmosphere with transmission coefficient ${\tilde{t}}_{12}\left(\omega \right)=t_{12}(\omega )e^{i\theta_{12}(\omega )}$ and reflection coefficient ${\tilde{r}}_{12}\left(\omega \right)=r_{12}(\omega )e^{i\varphi_{12}(\omega )}$. The transmitted wave constantly propagates until reflected from the ground plane. The complex propagation constant of the substrate layer can be written by $\tilde{\beta }=\beta_{1}+i\beta_{2}=\sqrt{\varepsilon_{d}}k_{0}d$, where $K_{0}$ is the free space wavenumber, $\beta_{1}$ is the propagation phase, absorption of the dielectric is represented by $\beta_{2}$, d is the substrate thickness, and $\varepsilon_{d}$ is the substrate dielectric constant. The entire wave is reflected due to the blocking of transmission by the ground layer after propagation phase delay $\tilde{\beta }$. The wave propagates toward the patch (layer 1) and is partially transmitted and reflected with transmission coefficient ${\tilde{t}}_{21}\left(\omega \right)=t_{21}(\omega )e^{i\theta_{21}(\omega )}$ and reflection coefficient ${\tilde{r}}_{21}\left(\omega \right)=r_{21}(\omega )e^{i\varphi_{21}(\omega )}$, respectively. Multiple reflection and transmission occur with the substrate, and total reflection is the superposition of all orders, which can be calculated by Equation (6). Figure 5b shows sound agreement between the IT model and CST-calculated absorption.

(6) $$\tilde{r}\left(\omega \right)={\tilde{r}}_{12}\left(\omega \right)-\frac{{\tilde{t}}_{12}\left(\omega \right){\tilde{t}}_{12}\left(\omega \right)e^{2i\tilde{\beta }}}{1+{\tilde{r}}_{21}\left(\omega \right)e^{2i\tilde{\beta }}}$$

$$A(\omega )=1-{\left|\tilde{r}(\omega )\right|}^{2}$$

The above calculation was performed for the normal incident angle. For oblique incident angle *θ*, the propagation phase delay was modified because the propagation length increases, $d^{\prime }=d/\cos\theta^{\prime }$, and the phase delay becomes $\tilde{\beta }=\sqrt{\varepsilon_{d}}k_{0}d^{\prime }$; the refractive angle *θ’* can be calculated by Snell’s law $\sqrt{\varepsilon_{d}}\sin\theta^{\prime }=\sin\theta$.

### 3.2. Polarization Conversion Ratio (PCR)

**Figure 6:**
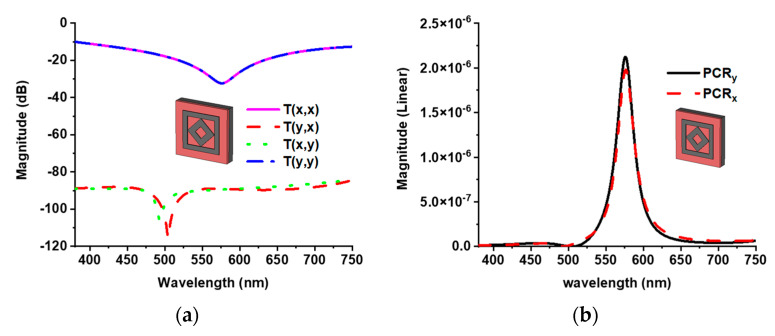
(**a**) Co- and cross-polarization component, and (**b**) PCR value of x and y polarization of the incident wave.

The PCR value of the proposed MMA calculated for verifying the proposed design is not a polarization converter but an absorber. Figure 6a shows the co- and cross-polarization as *Txx* and *Tyx* of x-polarization of the incident wave, respectively. The unique co- and cross-polarization of the x- and y-polarization of the incident wave were also achieved due to the structural symmetricity. The PCR value was calculated by Equations (7) and (8) for y and x polarization, respectively [35]. The PCR value was calculated as near-zero because of the very small cross-polarization *Tyx* = *Txy* < −90 dB. Figure 6b shows the PCR value of x- and y-polarized waves, which verify that the proposed design is an absorber, not a polarization converter.

(7) $$Y\;\mathrm{to}\;x\;\mathrm{polarized}\;\mathrm{wave},PCR_{y}=T_{xy}^{2}/\left(T_{yy}^{2}+T_{xy}^{2}\right)$$

(8) $$X\;\mathrm{to}\;y\;\mathrm{polarized}\;\mathrm{wave}\;PCR_{x}=T_{yx}^{2}/\left(T_{xx}^{2}+T_{yx}^{2}\right)$$

### 3.3. Metamaterial Property

**Figure 7:**
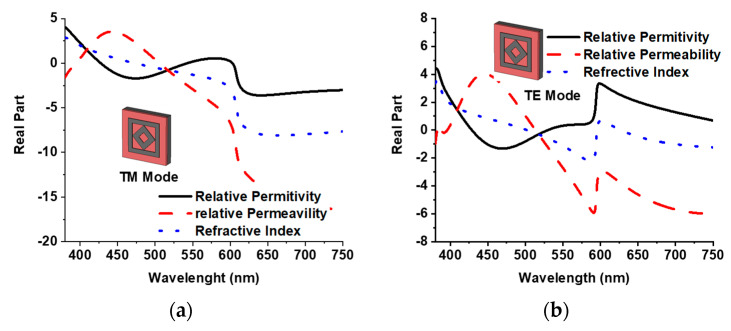
(**a**) Metamaterial property of TM mode, and (**b**) metamaterial property of TE mode.

Figure 7a,b present the metamaterial properties of the proposed MMA for TE and TM modes, respectively. In Figure 7a, the permittivity is negative from 427 to 541 nm and from 605 to 750 nm. The permeability is negative from 380 to 394 nm and from 506 to 750 nm. Both permeability and permittivity are negative from 505 to 540 nm and from 602 to 750 nm. A negative value of refractive index appears from 472 to 750 nm. As a result of this negative value of metamaterial property, the proposed MMA exhibits high-level broadband absorption properties in the TM mode. The TE mode alternatively achieves the negative value of metamaterial property, where a negative value of permeability is reached from 514 to 750 nm. A unique absorption curve is completed due to the structural symmetricity shown in Figure 4.

### 3.4. Polarization Insensitivity and Oblique Incident Angle Stability

**Figure 8:**
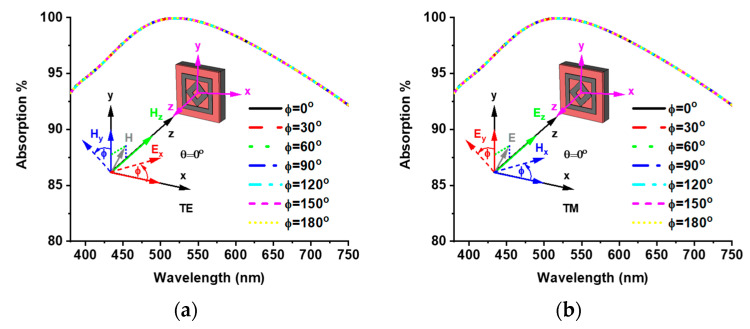
Polarization insensitivity of (**a**) TE mode and (**b**) TM mode.

**Figure 9:**
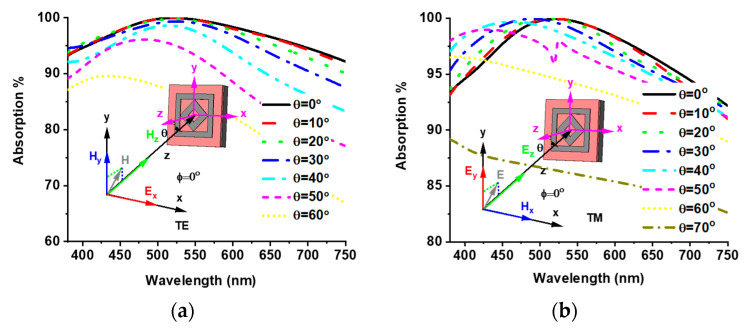
Oblique incident angle stability of (**a**) TE mode and (**b**) TM mode.

The polarization insensitivity of the proposed MMA was investigated to verify its absorption efficiency. Figure 8 shows the absorption property of different polarization incident angles (ф) for TE and TM modes. The TE mode z-axis is the direction of wave propagation, and the magnetic field vector Hz is along the z-axis. The electric field vector (Ex) and magnetic field vector (Hy) are along the x- and y-axis, respectively. On the other hand, in the TM mode, the electric field vector (Ez) is along the direction of wave propagation, and the magnetic (Hx) and electric (Ey) field vectors are along the x- and y-axis, respectively. Due to the axial and rotational symmetricity, the proposed MMA achieves unique absorption properties for polarization incident angles (ф) up to 180°. In all the above discussions, all results have been obtained from a normal incident angle (θ = 0°), but the EM wave occurs at an oblique incident angle on the MMA. Therefore, discussion of the absorption behavior of oblique incidents is also vital. The oblique incident angle (θ) is made between the direction of wave propagation and the z-axis of the MMA structure. Figure 9a,b show the absorption curve of both TE and TM modes for the oblique incident angles (θ) up to 60° and 70°, respectively, where an operating frequency above 70% absorption is found. Absorption properties of various oblique incident angles are listed in Table 2.

### 3.5. Bendable Property

**Figure 10:**
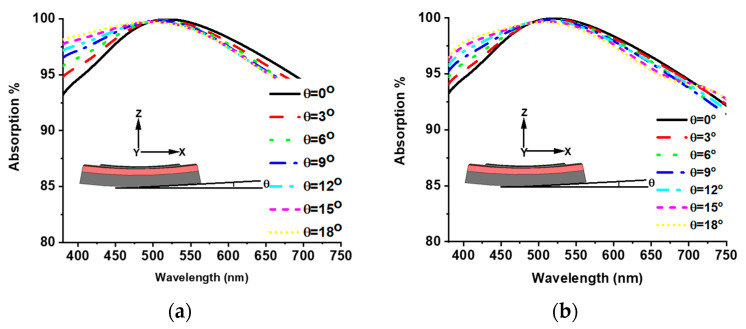
Bending effect of proposed MMA: (**a**) TE mode and (**b**) TM mode.

The MMA sheet may be curved due to the external effect during installation [36]. The banding effect on absorption behavior was investigated for both TE and TM modes. Figure 10 shows the absorption curve of banding angles θ = 0° to 18° with 3° intervals. In the TE mode, the absorption increases in the lower wavelength region and slightly decreases in the upper wavelength region with the increment in θ. The same absorption behavior is also shown in the TM mode. A unique absorption property is achieved for both TE and TM modes of various banding angles, which offers a bendable property of the proposed absorber.

### 3.6. Electric and Magnetic Field

**Figure 11:**
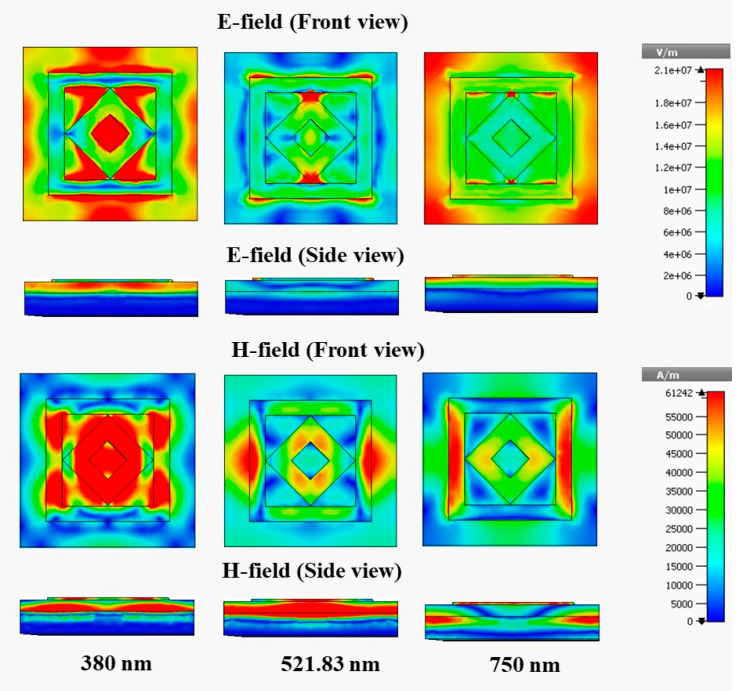
E-field and H-field distributions at TE mode.

**Figure 12:**
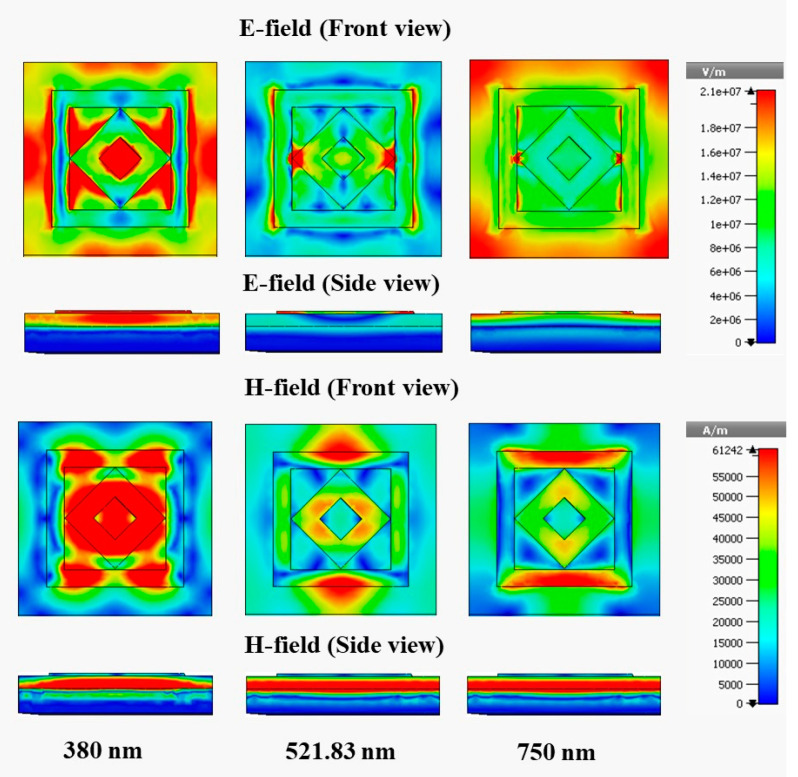
E-field and H-field distributions at TM mode.

Figure 11 and Figure 12 show the e-field (V/m) and h-field (A/m) distribution of the designed MMA for the normal incident angle θ = 0° of the TE and TM modes, respectively; the absorption mechanism can also be analyzed from this electromagnetic behavior. The electromagnetic property is enhanced and agitated at a different area of the absorber.

The e-field and h-field properties of 380 nm, 521.83 nm, and 750 nm are presented. The metamaterial property also significantly influences the electromagnetic behavior. The relationship between metamaterial properties and electromagnetic behavior can be understood from Equations (9) and (10).

(9) $$D=\varepsilon_{eff}\varepsilon_{0}E$$

(10) $$B=\mu_{eff}\mu_{0}H$$

where D is the electric flux density, B is the magnetic flux density, $\varepsilon_{eff}$ = effective permittivity, $\varepsilon_{0}$ = permittivity of free space, $\mu_{eff}$ = effective permeability, $\mu_{0}$ = permeability of free space, and E and H are the electric and magnetic field intensities, respectively. In the TE mode, at a wavelength of 380 nm, the maximum e-field is assimilated in the bounding area of the patch, where a h-field maximum intensity appears in the metal region of the patch. A high e-field intensity is also visible in the nonmetallic area of the dielectric medium, and the high magnetic field appears in the left and right arms of the outer ring. The e-field intensity is reduced at 750 nm compared with 350 nm, which shows the side view of the absorber and causes a lower 92.2% absorption at 750 nm compared with 94% absorption at 350 nm. The peak of 99.99% absorption is achieved at 521.83 nm, the e-field intensity is less than those at 380 and 750 nm, but a high h-field is created in the dielectric substrate, shown in the side view at 521.83 nm wavelength, and leads to an increased absorption peak. Figure 12 shows the electromagnetic behavior of the TM mode, where the field distribution is similar to that of the TE mode, but it rotates vertically to horizontal in the TM mode.

A comparison with the existing absorbers is presented in Table 3. The proposed SiO_2_ and W-based metamaterial absorber achieves 70° stability for an absorption above 70% with a wider operational bandwidth. An absorption above 92.2% and a peak absorption of 99.99% are also achieved. These excellent absorption results prioritize the proposed MMA over the listed absorber in Table 3.

## 4. Conclusions

This paper proposed a numerical analysis of a three-layer (W-SiO_2_-W) ultra-wide-band MMA for visible optical wavelength application. The projected design evaluation and geometrical parameters were investigated for achieving near-unity absorption, which was also validated by Interference Theory (IT). Analysis of the PCR value proved that the proposed model is an absorber, not a polarization converter. The absorption calculation of TE and TM modes was simultaneously presented, showing uniform absorption and increasing the acceptability of the proposed MMA. A peak absorption of 99.99% was achieved at 521.83 nm, and an absorption above 92.2% and an average absorption of 97.1% were achieved in the entire visible operational wavelength region. Both TE and TM modes showed polarization insensitivity, wide oblique incident angle stability, and sound absorption of 18° bending effects. These properties make the proposed MMA a potential candidate for various applications such as solar energy harvesting, light trapping, and optical sensors. Finally, a detailed comparison with existing MMAs showed the achieved unique features of the proposed MMA over the existing MMAs.

## Figures and Tables

**Table 1:** List of parameters of the proposed absorber.

Parameters	h1	h2	h3	W	W1	W2	W3
Value (nm)	120	62.82	15	950	670	350	150

**Table 2:** Absorption properties of various oblique incident angles.

Theta (θ)	TE Mode Absorption		TM Mode Absorption	
	Average	Peak	Average	Peak
0°	97.12%	99.99%	97.10%	99.99%
10°	97.07%	99.93%	97.07%	99.96%
20°	96.86%	99.70%	97.29%	99.99%
30°	96.02%	99.32%	97.51%	99.96%
40°	93.76%	98.60%	97.54%	99.70%
50°	90.61%	96.09%	96.81%	98.98%
60°	84.43%	89.61%	94.32%	96.64%
70°	70%	76.25%	86.41%	89.17%

**Table 3:** Performance comparison table of the proposed MMA.

Ref.	Material	Size (mm)	Operation Range	Bandwidth (nm)	Polarization Insensitivity	Incident Angle Stability A ≥ 70%	Absorption	Peak Absorption
[23]	Cu, Si_3_N_4_, Si	N/A	400–700	380	No	N/A	80%	97%
[24]	Au, SiO_2_	600 × 600 × 180	370–880	510	No	N/A	83%	92%
[25]	Ag, SiO_2_	350 × 350 × 180	400–700	300	No	N/A	80%	98%
[22]	W. SiO_2_	1000 × 1000 × 225	389.34–697.19	307.85	Yes	60°	91.24%	99.9%
[26]	Au, Si	500 × 500 × 600	474.4–784.4	310	Yes	65°	80%	98.5%
[27]	Ni, Si	250 × 250 × 355	400–700	300	Yes	60°	90%	99%
Proposed	W. SiO_2_	950 × 950 × 240.13	380–750	370	Yes	70°	92.2%	99.99%

## Data Availability

The data presented in this study are presented in this article.

## References

[1] Veselago V.G. (1968). The Electrodynamics of Substances with Simultaneously Negative Values of Img Align= Absmiddle Alt= ϵ Eps/Img and μ. Phys. Uspekhi.

[2] Shelby R.A., Smith D.R., Schultz S. (2001). Experimental Verification of a Negative Index of Refraction. Science.

[3] Liu J., Ma W.-Z., Chen W., Chen Y.-S., Deng X.-C., Gu Y. (2021). A Metamaterial Absorber Based on Particle Swarm Optimization Suitable for Earth’s Atmospheric Transparency Window. IEEE Access.

[4] Ren Z., Liu R., Zhang Y., Lu H., Li F., Liu Y., Hong X., Guo Y. (2021). Transmission reflection selective ultranarrow-band metamaterial filter based on electromagnetically induced transparency structure. Opt. Commun..

[5] Alam T., Almutairi A.F., Samsuzzaman M., Cho M., Islam M.T. (2021). Metamaterial array based meander line planar antenna for cube satellite communication. Sci. Rep..

[6] Lee Y.U., Posner C., Zhao J., Zhang J., Liu Z. (2021). Imaging of Cell Morphology Changes via Metamaterial-Assisted Photobleaching Microscopy. Nano Lett..

[7] Dash R., Sahu S., Mishra C., Sethi K., Palai G., Sahu S. (2016). Realization of ‘non-linear invisibility cloak’ using meta-material. Optik.

[8] Hakim M.L., Alam T., Almutairi A.F., Mansor M.F., Islam M.T. (2021). Polarization insensitivity characterization of dual-band perfect metamaterial absorber for K band sensing applications. Sci. Rep..

[9] Yu F.-Y., Shang X.-J., Fang W., Zhang Q.-Q., Wu Y., Zhao W., Liu J.-F., Song Q.-Q., Wang C., Zhu J.-B. (2022). A Terahertz Tunable Metamaterial Reflective Polarization Converter Based on Vanadium Oxide Film. Plasmonics.

[10] Tang W., Chen J., Cui T.J. (2021). Metamaterial Lenses and Their Applications at Microwave Frequencies. Adv. Photon. Res..

[11] Landy N.I., Sajuyigbe S., Mock J.J., Smith D.R., Padilla W.J. (2008). Perfect metamaterial absorber. Phys. Rev. Lett..

[12] Qiu Y., Zhang P., Li Q., Zhang Y., Li W. (2021). A perfect selective metamaterial absorber for high-temperature solar energy harvesting. Sol. Energy.

[13] Hamdy H., Abdel-Latif G.Y., El-Agamy M., El-Mikati H.A., Hameed M.F.O., Obayya S.S.A. (2022). Wavelength-selective metamaterial absorber based on 2D split rhombus grating for thermophotovoltic solar cell. Opt. Quantum Electron..

[14] Devine E.P. (2021). Mid-infrared photodetector based on 2D material metamaterial with negative index properties for a wide range of angles near vertical illumination. Appl. Phys. A.

[15] Cao T., Lian M., Lou X., Liu K., Guo Y., Guo D. (2021). Wideband mid-infrared thermal emitter based on stacked nanocavity metasurfaces. Int. J. Extreme Manuf..

[16] Huang L., Chen H.-T. (2013). A brief review on terahertz metamaterial perfect absorbers. Terahertz Sci. Technol..

[17] Zhu W. (2019). Electromagnetic Metamaterial Absorbers: From Narrowband to Broadband. Metamaterials and Metasurfaces.

[18] Mulla B., Sabah C. (2015). Perfect metamaterial absorber design for solar cell applications. Waves Random Complex Media.

[19] Ferraro A., Lio G.E., Hmina A., Palermo G., Djouda J.M., Maurer T., Caputo R. (2021). Tailoring of plasmonic functionalized metastructures to enhance local heating release. Nanophotonics.

[20] Lio G.E., Ferraro A., Giocondo M., Caputo R., De Luca A. (2020). Color Gamut Behavior in Epsilon Near-Zero Nanocavities during Propagation of Gap Surface Plasmons. Adv. Opt. Mater..

[21] Hossain M.J., Faruque M.R.I., Ahmed R., Alam J., Islam M.T. (2019). Polarization-insensitive infrared-visible perfect metamaterial absorber and permittivity sensor. Results Phys..

[22] Mahmud S., Islam S.S., Mat K., Chowdhury M.E.H., Rmili H., Islam M.T. (2020). Design and parametric analysis of a wide-angle polarization-insensitive metamaterial absorber with a star shape resonator for optical wavelength applications. Results Phys..

[23] Zhu P., Guo L.J. (2012). High performance broadband absorber in the visible band by engineered dispersion and geometry of a metal-dielectric-metal stack. Appl. Phys. Lett..

[24] Liu Z., Liu X., Huang S., Pan P., Chen J., Liu G., Gu G. (2015). Automatically Acquired Broadband Plasmonic-Metamaterial Black Absorber during the Metallic Film-Formation. ACS Appl. Mater. Interfaces.

[25] Butun S., Aydin K. (2014). Structurally tunable resonant absorption bands in ultrathin broadband plasmonic absorbers. Opt. Express.

[26] Hoa N.T.Q., Tung P.D., Lam P.H., Dung N.D., Quang N.H. (2018). Numerical Study of an Ultrabroadband, Wide-Angle, Polarization-Insensitivity Metamaterial Absorber in the Visible Region. J. Electron. Mater..

[27] Luo M., Shen S., Zhou L., Wu S., Zhou Y., Chen L. (2017). Broadband, wide-angle, and polarization-independent metamaterial absorber for the visible regime. Opt. Express.

[28] Ghosh G. (1999). Dispersion-equation coefficients for the refractive index and birefringence of calcite and quartz crystals. Opt. Commun..

[29] Ding J., Feng A., Li X., Ding S., Liu L., Ren W. (2021). Properties, preparation, and application of tungsten disulfide: A review. J. Phys. D Appl. Phys..

[30] Kanmaz I., Abdullah Ü. (2021). Silicon dioxide thin films prepared by spin coating for the application of solar cells. Int. Adv. Res. Eng. J..

[31] AGDC (2019). CST Studio Suite. https://www.3ds.com/products-services/simulia/products/cst-studio-suite/..

[32] Cen C., Chen Z., Xu D., Jiang L., Chen X., Yi Z., Wu P., Li G., Yi Y. (2020). High quality factor, high sensitivity metamaterial graphene—Perfect absorber based on critical coupling theory and impedance matching. Nanomaterials.

[33] Hakim M.L., Alam T., Soliman M.S., Sahar N.M., Baharuddin M.H., Almalki S.H.A., Islam M.T. (2022). Polarization insensitive symmetrical structured double negative (DNG) metamaterial absorber for Ku-band sensing applications. Sci. Rep..

[34] Mahmud S., Karim M., Islam S.S., Shuvo M.K., Akter T., Almutairi A.F., Islam M.T. (2021). A Multi-Band Near Perfect Polarization and Angular Insensitive Metamaterial Absorber With a Simple Octagonal Resonator for Visible Wavelength. IEEE Access.

[35] Xiao Z.-Y., Liu D.-J., Ma X.-L., Wang Z.-H. (2015). Multi-band transmissions of chiral metamaterials based on Fabry-Perot like resonators. Opt. Express.

[36] Deng B., Xu R., Zhao K., Lu Y., Ganguli S., Cheng G.J. (2018). Composite bending-dominated hollow nanolattices: A stiff, cyclable mechanical metamaterial. Mater. Today.

